# Uterine Torsion: A Rare Condition in a Nullipara Woman

**DOI:** 10.7759/cureus.48751

**Published:** 2023-11-13

**Authors:** Ruhida Razzak, Shila Shelke, Poonam V Shivkumar

**Affiliations:** 1 Obstetrics and Gynaecology, Mahatma Gandhi Institute of Medical Sciences, Wardha, IND

**Keywords:** myomectomy, nullipara, leiomyoma, uterine torsion, subserosal fibroid

## Abstract

Uterine torsion, an infrequent entity, is defined as the rotation of the uterus greater than 45° around the longitudinal axis of the uterus. It is usually found in a gravid uterus being extremely uncommon in nulliparas. Here, we are presenting a case of a 22-year-old woman who presented with complaints of constant, dull aching pain in the abdomen with a palpable huge mass. An emergency laparotomy was done revealing a large 12 x 10 x 8 cm large subserosal fibroid and the uterus rotated on its own axis to about 180 degrees with bilaterally enlarged cystic ovaries. Derotation and subsequent myomectomy were done. The weight of the subserosal fibroid caused the uterus to rotate on its own axis. As it is a rare entity, a high level of suspicion and timely surgical intervention is the need of the hour to prevent further morbidity and mortality.

## Introduction

The uterus rotates more than 45 degrees around its long axis [1]. Animal cases of uterine torsion are frequently documented. While being unusual, it has been recorded in women of all ages, commencing before the onset of menses to post-menopausal phases. Most cases affect pregnant women, and non-pregnant women remain rather rarely affected [2,3]. Pregnancy amplifies the congenital and physiologic rotations and obliquities of the uterus, thus explaining why the torsion of the uterus occurs more usually in the gravid uterus compared to the non-gravid uterus [1,4]. It has been seen in normal pregnancies and typically happens in the third trimester, having serious maternal and perinatal implications. A torsion in a pregnant uterus is linked to a high perinatal mortality rate of about 12% of cases [5]. As it is an uncommon entity in a nullipara with vague symptoms, diagnosing it can be challenging. It can also be fatal and contribute to infertility. Thus, there is a need for rapid surgical intervention and a high level of clinical suspicion.

## Case presentation

A 22-year-old, young woman, unmarried nullipara, presented to the emergency department of our rural hospital with complaints of severe pain in the abdomen. She had started to experience swelling over her lower abdomen for one month accompanied by on-and-off pain in the abdomen, relieved by taking over-the-counter painkillers but her pain increased drastically three to four hours before presentation. The patient had attained menarche at the age of 14 years. She had regular menstrual cycles of 26-28 days lasting for four days with average flow, changing two to three pads per day, not associated with clots and dysmenorrhea. Her past medical, surgical, and family history were insignificant. 

On examination, a hard mass was palpated with the mass corresponding to almost 16 weeks of pregnancy with tenderness at the right iliac fossa. A urine pregnancy kit was done at the bedside, which was negative. This was done as a high number of unmarried females hide the fact of being pregnant as it is considered taboo in this rural area. She was started on injectable analgesics for pain relief. Her tenderness increased from the previous examination with no relief in pain with increased pulse rate with dropping blood pressure. Due to the non-availability of a portable ultrasonography machine in the emergency room as the hospital was situated in a rural setup, the radiological investigation could not be carried out. Within 35-45 minutes of her admission, a decision was taken to shift her for an emergency laparotomy.

**Figure 1:**
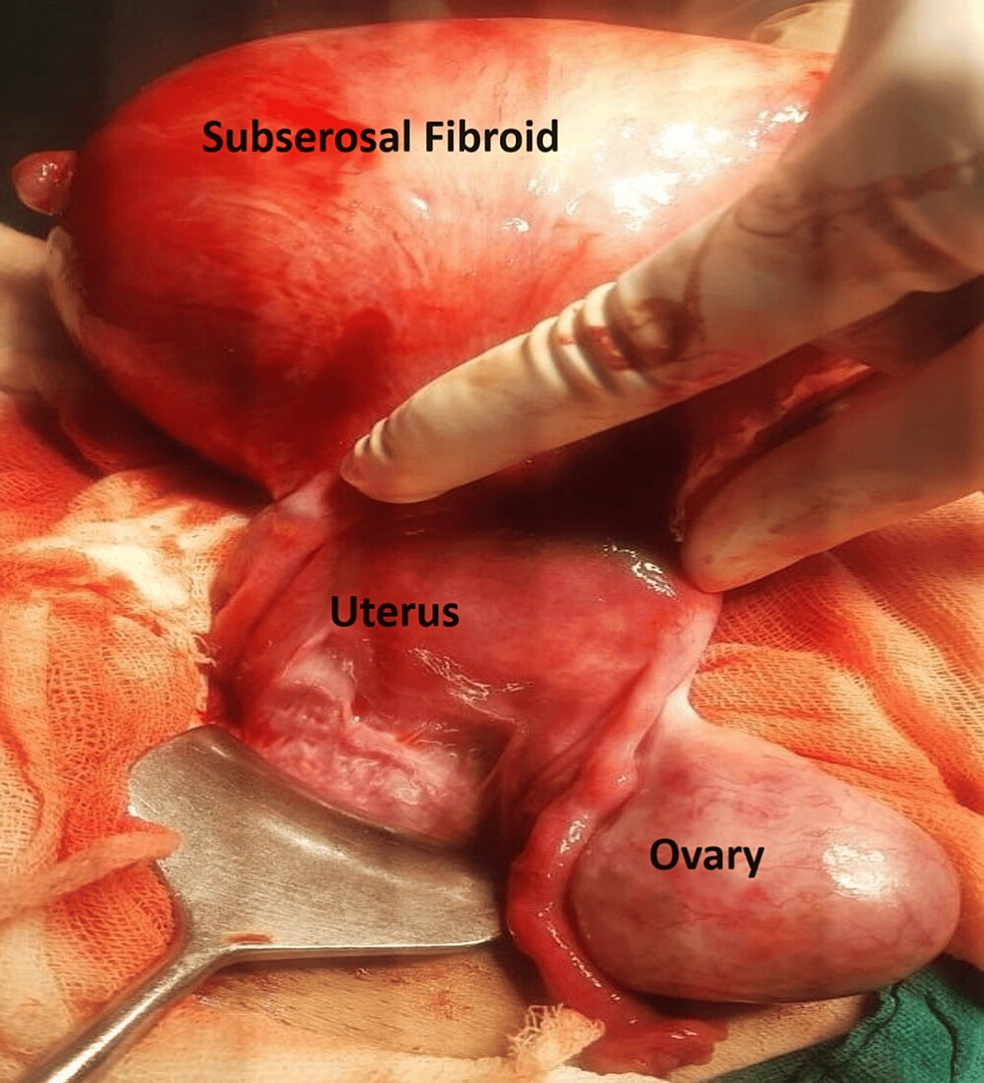
Figure showing torsion of uterus, large subserous fibroid, and large bulky ovary

The abdomen was opened by a suprapubic transverse incision. Intraoperatively, a large sub-serosal fibroid of around 15x12x12 cm with multiple small fibroids of 1x1 cm was seen arising above the fundus, with the uterus dextrorotated to around 180 degrees, with large cystic bilateral ovaries (Figure 1). 

**Figure 2:**
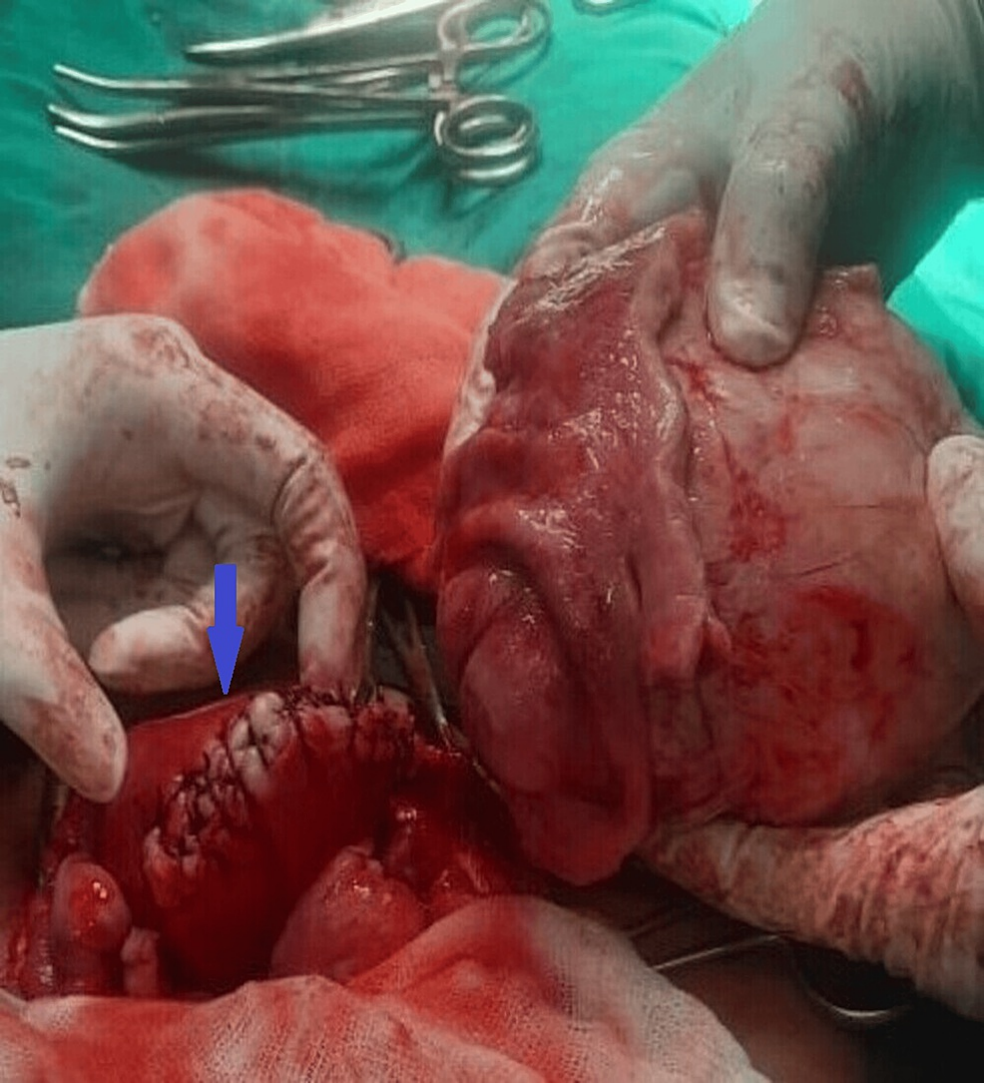
Baseball sutures over fundus

Firstly, dextrorotation was corrected. Then the subcapsular plain of the subserosal fibroid was infiltrated with 1:200 concentration of vasopressin in normal saline, and the whole of the mass was excised in toto. We did not enter the uterine cavity. Fallopian tubes were patent and no cornual structures were injured while taking out the fibroid. The base of the fundal part of the uterus was sutured with baseball sutures in a double layer (Figure 2).

**Figure 3:**
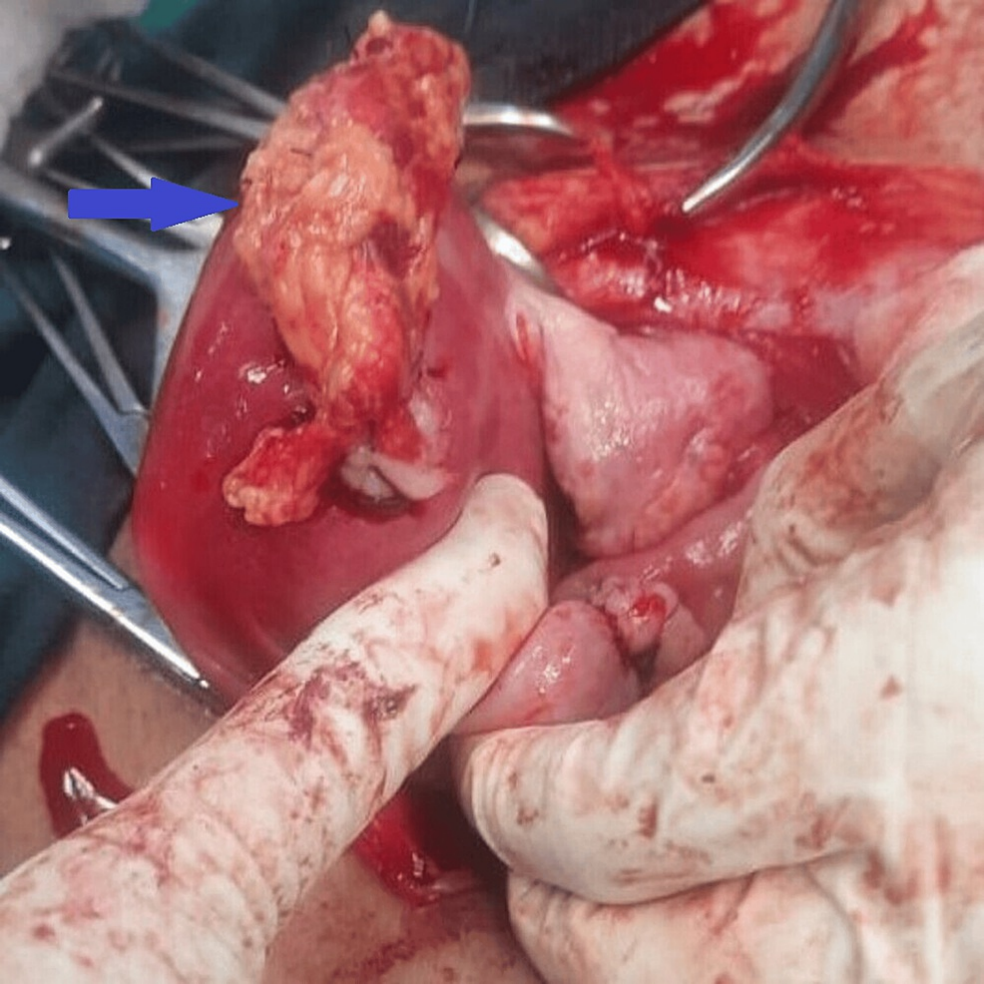
Omental patch over suture line

The omental patch was mobilized and attached to the suture line so as to prevent adhesion formation (Figure 3).

As ovaries were large, cystic, and bulky, we needed to prevent the future development of polycystic ovarian syndrome. After obtaining informed consent from the patient's mother, we cauterized the bulky, cystic ovaries at four sides at around a depth of four millimeters for four seconds. Right-sided round ligament plication was done so as to prevent retorsion. The abdomen was closed in layers. Her histopathology report suggested that the mass was a large leiomyoma with hyaline changes.

Postoperatively, the patient was stable and recovered uneventfully. She was put on injectable antibiotics ceftriaxone and metronidazole along with injectable acetaminophen for three days. She resumed her day-to-day activities from day three onwards. Her dressing was done on postoperative day three and day five and complete suture removal was done on day eight, which was healthy. She attended her follow-up visits regularly after her discharge from the hospital. She and her parents were counseled about the remote complication regarding future pregnancy and was advised to get registered at a hospital when she conceives keeping regular follow-up.

## Discussion

Uterine torsion in a non-gravid woman is extremely rare. Only 25 cases of uterine torsion in non-gravid women have been reported in the English literature available on PubMed over the last 20 years. The first two cases were described by Virchow in 1861 and later in 1863 in combination with the uterine fibroids during the autopsies [6]. In two-thirds of cases, rotation takes towards the right. The torsion usually takes place near the transition zone of the corpus with cervix uteri. Torsion is usually prevented by the round and broad ligaments. It is not clearly understood why torsion takes place but it is found that usually there is a pathological or abnormal condition of the uterus or its adjacent organ [7].

Abdominal injuries, congenital abnormalities, and uterine masses have been reported to be risk factors for uterine torsion. Due to its rare and asymptomatic nature, uterine torsion is typically identified during surgery, despite the fact that CT, MRI, and ultrasonography have shown potential in preoperative diagnosis. The preferred method of treatment is laparotomy and detorsion. CT scans and MRIs frequently reveal characteristic findings, specifically the "whirl sign" of the uterine cervix, which may be valuable for early diagnosis [8]. Patients with uterine torsion may experience irreversible ischemia and eventual necrosis, highlighting the significance of timely radiological imaging. Torsion may occur in a number of additional nearby organs, including the spleen, ovaries, and fallopian tubes.

In this case, where a young, unmarried female presented with an acute abdomen with a large palpable mass, it was important to rule out other differential causes of acute abdomen along with pregnancy, acute appendicitis, renal stones, or a case of torsion of ovary. As the diagnosis of uterine torsion in a nulligravida is rare and termed as a ‘once in a lifetime diagnosis’, its suspicion was also not raised in the differentials. In a rural setup like our hospital, where a portable ultrasonography machine is not available, screening ultrasonography could not be done before shifting the patient to the operating room. It was quite surprising to find the intra-operative findings. As the fundal part of the uterus was handled during our intervention, special instructions were given to the patient regarding her future pregnancies, which included getting registered at a tertiary care center when pregnant where proper monitoring can take place so as to rule out a possibility of scar dehiscence or rupture. Regular fortnightly or weekly follow-up after 28 weeks of gestation is recommended in such cases.

## Conclusions

In non-pregnant women, uterine torsion is uncommon and thus remains frequently undiagnosed, which can lead to catastrophic consequences. In non-gravid women, uterine torsion is typically associated with non-specific symptoms and laboratory data according to a review of recent cases. Thus, when a woman of any age appears with acute abdominal discomfort, medical professionals should consider uterine torsion as a differential diagnosis. The case presented in this report serves as an example of how uterine torsion should be taken into account when women with pelvic tumors complain of sudden abdominal pain.
